# Supplementary material for: Effects of SKCPT on Osteoarthritis in Beagle Meniscectomy and Cranial Cruciate Ligament Transection Models
Source: Int J Mol Sci. 2023 Oct 7;24(19):14972. doi: 10.3390/ijms241914972 (PMC10573642; doi:10.3390/ijms241914972)
Supplement: Supplementary file 1 [file ijms-24-14972-s001.zip › ijms-2610633-supplementary.pdf]

Supplementary Figure & Tables

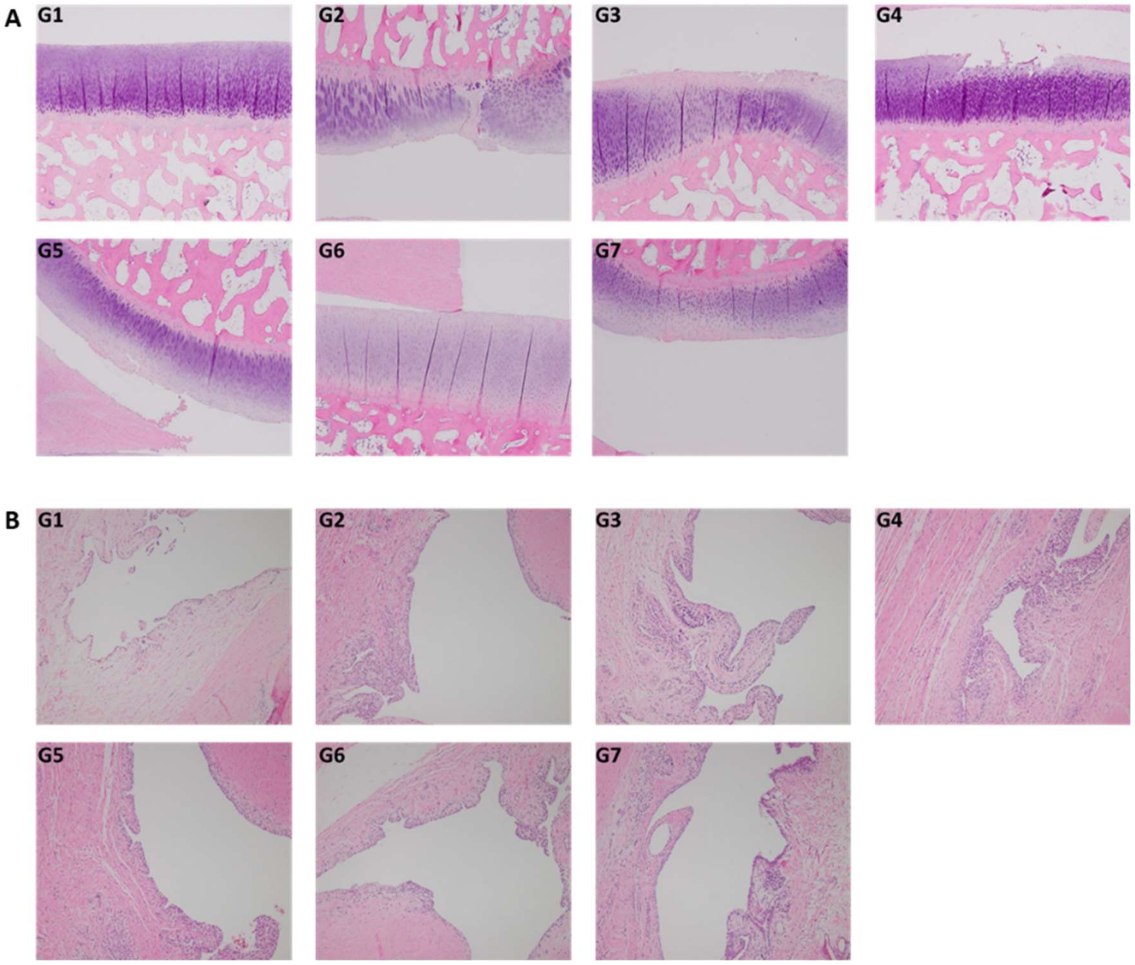

Figure S1. The results of histological examination in a canine model of OA. The paraffin-embedded tissue slides of cartilage or synovium were stained with H&E dye. For microscopic examination, a magnification of X40 was utilized for (A) cartilage, while a magnification of X100 was employed for (B) synovium. The total cartilage score and total synovial score were quantified and subsequently incorporated into the OARSI score. Data are expressed as Mean ± S.D. Day of first test article administration was designated day 0. G1: Normal control, G2: Vehicle control, G3: Positive control, G4: 200 mg IR, G5: 300 mg IR, G6: 300 mg MR, G7: CR-1

Table S1. The criteria of gait evaluation.

Score	Parameters
0	No observable lameness
1	Intermittent, mild weight-bearing lameness with little, if any, change in gait
2	Moderate weight-bearing lameness–obvious lameness with noticeable gait change
3	Severe weight-bearing lameness– "toe-touching" only
4	Non-weight-bearing

Table S2. Grading of cartilage structure.

Severity of Pathology Characteristics		Area of Section Affected			
		None	Local (Approx 1/3)	Multi-Focal (Approx 2/3)	Global (>2/3)
A	Normal volume, smooth surface with all zones intact	0	0	0	0
B	Surface undulations including fissures in surface/upper zone and/or pannus tissue formation on surface	0	1	2	3
C	Fissures to mid-zone and/or erosion of surface/upper zone	0	2	4	6
D	Fissures that extend to deep zone and/or erosion through mid-zone	0	3	6	9
E	Full thickness loss of cartilage	0	4	8	12

Table S3. Grading of chondrocyte pathology.

Severity of Pathology Characteristics		Area of Section Affected			
		None	Local (Approx 1/3)	Multi-Focal (Approx 2/3)	Global (>2/3)
A	Normal	0	0	0	0
B	Loss of cells in the surface zone or relative increased density with occasional superficial clusters	0	1	2	3
C	Small cell clusters (2–4 cells/cluster) predominate	0	2	4	6
D	Large cell clusters (≥5 cells/cluster) predominate	0	3	6	9
E	Cell loss (necrosis/apoptosis) predominates	0	4	8	12

Table S4. Grading of proteoglycan staining.

Severity of Pathology Characteristics		Area of Section Affected			
		None	Local (Approx 1/3)	Multi-Focal (Approx 2/3)	Global (>2/3)
A	Normal	0	0	0	0
B	Decreased proteoglycan content in surface/upper zone	0	1	2	3
C	Decreased proteoglycan content into the mid-zone	0	2	4	6
D	Decreased proteoglycan content into the deep zone	0	3	6	9
E	Full depth decrease in proteoglycan content	0	4	8	12

Table S5. Grading of lining cells characteristics.

Severity of Pathology Characteristics		Area of Section Affected			
		None	Local (Approx 1/3)	Multi-Focal (Approx 2/3)	Global (>2/3)
A	1–2 layers of cells	0	0	0	0
B	3–6 layers of cells	0	1	2	3
C	>6 layers of cells	0	2	4	6

Table S6. Grading of cell infiltration characteristics.

Severity of Pathology Characteristics		Area of Section Affected			
		None	Local (Approx 1/3)	Multi-Focal (Approx 2/3)	Global (>2/3)
A	No cellular infiltration	0	0	0	0
B	Mild to moderate inflammatory cell infiltrates including small lymphoid follicles	0	1	2	3
C	Marked, diffuse inflammatory cell infiltrates including large lymphoid follicles	0	2	4	6

Table S7. Grading of hyperplasia characteristics.

Severity of Pathology Characteristics		Area of Section Affected			
		None	Local (Approx 1/3)	Multi-Focal (Approx 2/3)	Global (>2/3)
A	No villous hyperplasia	0	0	0	0
B	Short villi	0	1	2	3
C	Finger-like hyperplasia	0	2	4	6
